# Wogonin, a Compound in *Scutellaria baicalensis*, Activates ATF4–FGF21 Signaling in Mouse Hepatocyte AML12 Cells

**DOI:** 10.3390/nu14193920

**Published:** 2022-09-21

**Authors:** Yasunari Yamada, Hodaka Saito, Masaya Araki, Yuhei Tsuchimoto, Shin-ichi Muroi, Kyohei Suzuki, Kazufumi Toume, Jun-Dal Kim, Takashi Matsuzaka, Hirohito Sone, Hitoshi Shimano, Yoshimi Nakagawa

**Affiliations:** 1 Division of Complex Biosystem Research, Department of Research and Development, Institute of Natural Medicine, University of Toyama, Toyama 930-0194, Japan; 2 Department of Endocrinology and Metabolism, Faculty of Medicine, University of Tsukuba, Tsukuba 305-8575, Japan; 3 Section of Pharmacognosy, Institute of Natural Medicine, University of Toyama, Toyama 930-0194, Japan; 4 Life Science Center for Survival Dynamics, Tsukuba Advanced Research Alliance (TARA), University of Tsukuba, Tsukuba 305-8577, Japan; 5 Transborder Medical Research Center (TMRC), University of Tsukuba, Tsukuba 305-8575, Japan; 6 Department of Hematology, Endocrinology and Metabolism, Niigata University Faculty of Medicine, Niigata 951-8510, Japan; 7 International Institute for Integrative Sleep Medicine (WPI-IIIS), University of Tsukuba, Tsukuba 305-8575, Japan; 8 Japan Agency for Medical Research and Development-Core Research for Evolutional Science and Technology (AMED-CREST), Tokyo 100-0004, Japan

**Keywords:** wogonin, fibroblast growth factor, activating transcription factor 4, WAKANYAKU, *Scutellaria baicalensis*

## Abstract

Fibroblast growth factor 21 (FGF21), which is mainly synthesized and secreted by the liver, plays a crucial role in systemic glucose and lipid metabolism, ameliorating metabolic diseases. In this study, we screened the WAKANYAKU library derived from medicinal herbs to identify compounds that can activate *Fgf21* expression in mouse hepatocyte AML12 cells. We identified *Scutellaria baicalensis* root extract and one of its components, wogonin, as an activator of *Fgf21* expression. Wogonin also enhanced the expression of activating transcription factor 4 (ATF4) by a mechanism other than ER stress. Knockdown of ATF4 by siRNA suppressed wogonin-induced *Fgf21* expression, highlighting its essential role in wogonin’s mode of action. Thus, our results indicate that wogonin would be a strong candidate for a therapeutic to improve metabolic diseases by enhancing hepatic FGF21 production.

## 1. Introduction

Fibroblast growth factor 21 (FGF21) is expressed in various tissues including the liver, pancreas, brown adipose tissue (BAT), and white adipose tissue (WAT) [1]. The receptor for FGF21 forms complexes. The FGF21 receptor complex comprises FGF receptor 1c (FGFR1c) and the co-factor β-Klotho [2,3]. β-Klotho is highly expressed in the liver, gall bladder, colon, pancreas, BAT, and WAT [1]. FGFR1c is widely expressed, but little or not expressed in the liver [1]. Thus, the effects of FGF21 on the liver are thought to be indirect. Under normal conditions, plasma FGF21 is secreted from the liver [4]. FGF21 is mainly secreted into the bloodstream by the liver and affects various peripheral tissues to normalize systemic glucose and lipid metabolism. FGF21 reduces plasma glucose levels by increasing glucose uptake by adipose tissues [5]. It also induces thermogenic gene expression and browning in the white adipose tissue by increasing the levels of peroxisome proliferator-activated receptor (PPAR)-γ coactivator-1α (PGC-1α). Hence, FGF21 knockout mice showed impaired adaptation to cold exposure [6]. FGF21 has been shown to normalize plasma glucose, insulin, and triglyceride levels in some type 2 diabetes mouse models [5,7]. Thus, it is a therapeutic target for metabolic diseases.

Hepatic gene expression of FGF21 was up-regulated in response to fasting by PPARα [8] and cyclic adenosine monophosphate-responsive element-binding protein H (CREBH) [9]. Other transcription factors that activate Fgf21 expression include activating transcription factor 4 (ATF4) [10], ATF6 [11], carbohydrate-responsive element-binding protein (ChREBP) [12], nuclear factor-like 2 (NRF2) [13], and X-box binding protein 1s (XBP1s) [14].

ATF4 is a basic leucine zipper domain-containing transcription factor that regulates a gene expression program in the integrated stress response (ISR), including the activation of autophagy during amino acid deprivation [15,16], the stimulation of anti-oxidant defenses during oxidative stress [17], and the inhibition of mRNA translation and elevation of protein folding capacity during endoplasmic reticulum (ER) stress [18]. The accumulation of unfolded proteins in the ER causes ER stress, which activates the ISR [19]. ATF4 activates gene expression, including that of *Fgf21*, by directly binding to amino acid response element sequences in the promoter region of its target genes [20]. The expression of ATF4 is up-regulated by NRF2 [21], transcription factor E3 (TFE3), and TFEB [22], while it is down-regulated by CCAAT/enhancer-binding protein β (C/EBPβ) [23]. The translation of ATF4 is activated by double-stranded RNA-dependent protein kinase (PKR)-like endoplasmic reticulum kinase (PERK)-phosphorylated eukaryotic initiation factor 2α (eIF2α) [20]. The role of ISR in metabolic diseases is dichotomous. The ISR induces hepatic steatosis. On the other hand, ISR induces *Fgf21* expression [10,24], which improves hepatic steatosis and glucose intolerance [25,26].

The crude drug *Scutellaria baicalensis* root (Scutellaria root) is widely used as a traditional oriental medicine. *S. baicalensis* root extract (SBE) has antioxidant [27], antitumor [28], anti-inflammatory [29], antiviral [30], and neuroprotective effects [31]. SBE has been shown to ameliorate non-alcoholic fatty liver disease [32] and diabetes [33,34]. All these effects are due to the flavonoids in SBE, chiefly, wogonin, baicalin, and baicalein [35]. Wogonin counters hyperglycemia and hyperlipidemia in *db/db* mice by stimulating PPARα and adiponectin expression by activating adenosine monophosphate (AMP)-activated protein kinase (AMPK) in adipose tissue [36]. Wogonin activates PPARα and adiponectin receptor 2 in the liver of diet-induced obesity mice, ameliorating the metabolic disorder [37].

In this study, we screened natural compounds from the Natural Medicine (WAKANYAKU) library that can activate *Fgf21* expression in mouse hepatocyte AML12 cells. We identified that SBE and one of its components, wogonin, activates *Fgf21* expression. We found that wogonin is a potential compound to activate *Fgf21* expression, mediated by ATF4 in AML12 cells.

## 2. Materials and Methods

### 2.1. Chemicals

The WAKANYAKU library consisted of 122 extracts of crude drugs (Table S1), which were provided by the Institute of Natural Medicine, University of Toyama. All crude drugs were purchased from Tochimoto Tenkaido Co., Ltd (Osaka, Japan). The voucher specimens of these crude drugs were deposited in the Museum of Materia Medica, Institute of Natural Medicine (TMPW), University of Toyama. The 30.0 g of each crude drug was extracted with purified water (300 mL) by boiling for 60 min. The filtrated decoction was lyophilized to obtain dry extract powder. Each extract was dissolved in water (10 mg/mL). Wogonin (TOKYO CHEMICAL INDUSTRY CO., LTD, Tokyo, Japan, W0010), baicalin (Combi-Blocks, San Diego, CA, USA, QB-9653), and baicalein (BLDpharm, Shanghai, China, BD6296) were purchased.

### 2.2. Cell Culture

AML12 cells were cultured at 37 °C in a 5% CO_2_ environment in D-MEM/Ham’s F-12 medium (WAKO, Osaka, Japan, 048-29785) supplemented with 10% fetal bovine serum (CORNING, NY, USA, 35-079-CV), 100 U/mL penicillin, 100 μg/mL streptomycin (Nacalai Tesque, Kyoto, Japan), and 1% ITS-G Supplement (WAKO, 090-06741). Cells were treated with 10, 50, or 100 µg/mL of SBE and 10 or 20 µM of wogonin, baicalin, and baicalein.

### 2.3. Plasmids and Small Interfering RNA (siRNA)

pGL3-FGF21 contained –2 kbp to –40 bp of the mouse *Fgf21* promoter [9]. pGL3-ATF4 contained –0.5 kbp to –100 bp of the mouse *Atf4* promoter. pRK-ATF4, the human ATF4 expression vector, was a gift from Yihong Ye (Addgene plasmid #26114) [38]. siRNAs against luciferase (siLuc) (Invitrogen, Waltham, MA, USA, 12935-146) and ATF4 (Santa Cruz, Dallas, TX, USA, sc-35113) were purchased. These plasmids and siRNAs were transfected into AML12 cells with Lipofectamine 3000 (Thermo Fisher Scientific, Waltham, MA, USA).

### 2.4. Screening Analysis to Increase Fgf21 Expression Using the WAKANYAKU Library

AML12 cells were transfected with pGL3-FGF21 and pRL-CMV (Promega, Madison, WI, USA), as a reference, with Lipofectamine 3000 (Thermo Fisher Scientific). After 24 h of transfection, each crude drug from the WAKANYAKU library was added to the medium at 10 µg/mL. After an additional 24 h incubation, cells were collected. Firefly and renilla luciferase activity were measured using the Dual-Luciferase^®^ Reporter Assay System (Promega). Firefly luciferase activities were normalized to renilla luciferase activities.

### 2.5. Luciferase Analysis

AML12 cells were transfected with the indicated luciferase vector and pRL-CMV (Promega), as a reference, using Lipofectamine 3000 (Thermo Fisher Scientific). After a 24 h incubation, cells were treated with the indicated concentrations of flavonoids for 24 h. Firefly and renilla luciferase activity was measured using the Dual-Luciferase^®^ Reporter Assay System (Promega). Firefly luciferase activities were normalized to renilla luciferase activities.

### 2.6. Quantitative Polymerase Chain Reaction (qPCR)

Total RNA was isolated from collected cells using Sepasol^®^-RNA I Super G (Nacalai Tesque, 09379-55) according to the manufacturer’s protocol. All samples passed the RNA quality control as assessed on the NanoDrop 1000 Spectrophotometer. cDNA was generated using the PrimeScript™ RT Master Mix (Perfect Real Time) (Takara Bio, Kusatsu, Japan, RR036). qPCR was performed on a CFX Connect Real-Time PCR Detection System (Bio-Rad, Hercules, CA, USA) using TB Green^®^ Premix Ex Taq™ II (Tli RNaseH Plus) (Takara Bio. RR820) or THUNDERBIRD^®^ Next SYBR^®^ qPCR Mix (Toyobo, Osaka, Japan, QPX-201). Samples were quantified by the ΔΔC_t_ method and normalized to *Cyclophilin* levels to quantify the relative mRNA expression. qPCR primer sequences are listed in Table 1.

### 2.7. Western Blotting

Cells were lysed in the lysis buffer containing 50 mM HEPES, 200 mM NaCl, 1% NP-40, 100 mM NaF, 0.5% sodium pyrophosphate, 10% glycerol, and cOmplete protease inhibitor (Roche, Basel, Switzerland). The total cell lysates were subjected to sodium dodecyl sulfate-polyacrylamide gel electrophoresis (SDS-PAGE) and transferred to Immobilon-P PVDF membranes (Millipore, Burlington, MA, USA). The membranes were incubated with anti-ATF4 (Santa Cruz, sc-390063, 1:000), anti-phosho-eIF2α (Cell Signaling Technologies, Danvers, MA, USA, 3298, 1:1000), anti-eIF2α (Cell Signaling Technologies, 5324, 1:1000), and anti-GAPDH (WAKO, 016-25523, 1:5000) antibodies. GAPDH was used as an internal control. After washing, the membranes were incubated with horseradish peroxidase-conjugated mouse IgG (Cell Signaling Technologies, 7076, 1:5000) and rabbit IgG (Cell Signaling Technologies, 7074, 1:5000). The immunoreactive bands were detected by ChemiDoc XRS+ (BioRad) using ImmunoStar LD (WAKO, 290-69904). The intensity of immunoreactive bands was quantified with the Image Lab software (BioRad).

### 2.8. Statistical Analyses

All data were expressed as the mean ± standard deviation (SD). Statistical significance between the two groups was calculated with the unpaired Student’s t-test. Statistical significance among multiple groups was calculated with one-way ANOVA, followed by Tukey’s post hoc test using GraphPad Prism 7 (GraphPad Prism software, San Diego, CA, USA). *p*-values < 0.05 were considered statistically significant.

## 3. Results

### 3.1. Cell-Based Screening Using a Natural Medicine Library Identified That SBE Induced Fgf21 Expression

**Figure 1:**
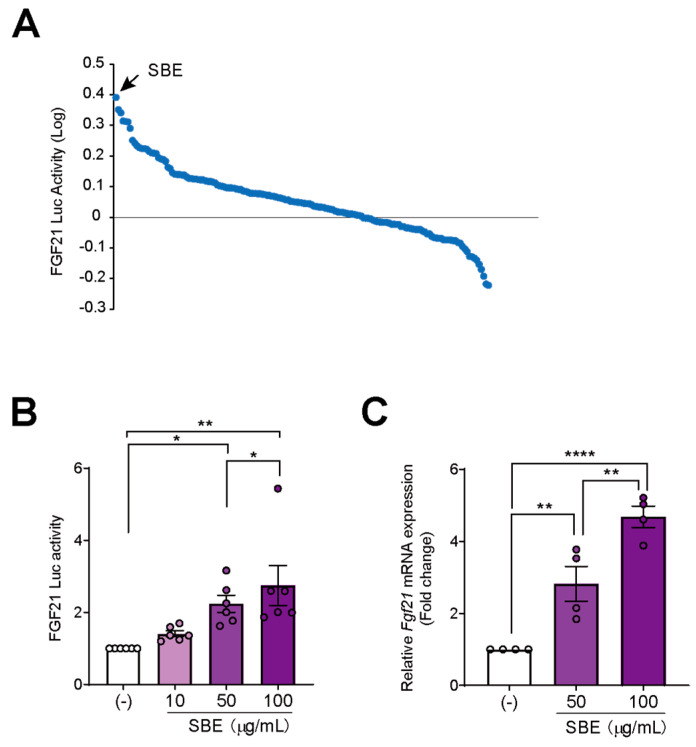
SBE is identified as an activator of *Fgf21* expression after screening the WAKANYAKU library. (**A**) The screening of natural medicines from the WAKANYAKU library that can induce *Fgf21* expression using an FGF21–luciferase assay. AML12 cells were co-transfected with the reporter vector pGL3-FGF21 and pRL-SV40 as a reference. After 24 h of transfection, cells were treated with 10 µg/mL of natural medicines for 24 h. The luciferase activity was measured and normalized to the renilla luciferase activity. (**B**) SBE activated FGF21-luciferase activity in AML12 cells. Cells were co-transfected with pGL3-FGF21 and pRL-SV40 vectors. After 24 h of transfection, cells were treated with 10, 50, 100 µg/mL of SBE for 24 h. *n* = 4 per group. (**C**) SBE increased *Fgf21* expression in AML12 cells. Cells were treated with 50 and 100 µg/mL of SBE for 48 h. *n* = 4 per group. Data are represented as mean ± SD. * *p* < 0.05; ** *p* < 0.01; **** *p* < 0.0001. Comparisons among multiple groups were assessed using one-way ANOVA, followed by Tukey’s post hoc test.

To identify novel activators of *Fgf21* expression, we screened 122 crude drugs contained in the WAKANYAKU library by performing the luciferase assay on pGL3-FGF21-transfected AML12 cells after treating them with 10 µg/mL of each drug for 24 h. SBE increased FGF21-luciferase activity to the greatest extent (Figure 1A). To confirm the stimulatory effects of SBE, we performed a dose-dependent experiment and found that FGF21-luciferase activity increased with the dose of SBE (Figure 1B). To further confirm the effects of SBE on *Fgf21* expression, we performed qPCR analysis. SBE also induced *Fgf21* mRNA expression in AML12 cells in a dose-dependent manner (Figure 1C). Taken together, we identified SBE to be a novel activator of *Fgf21* expression.

### 3.2. Wogonin, a Flavonoid in SBE, Induces Fgf21 Expression in AML12 Cells

**Figure 2:**
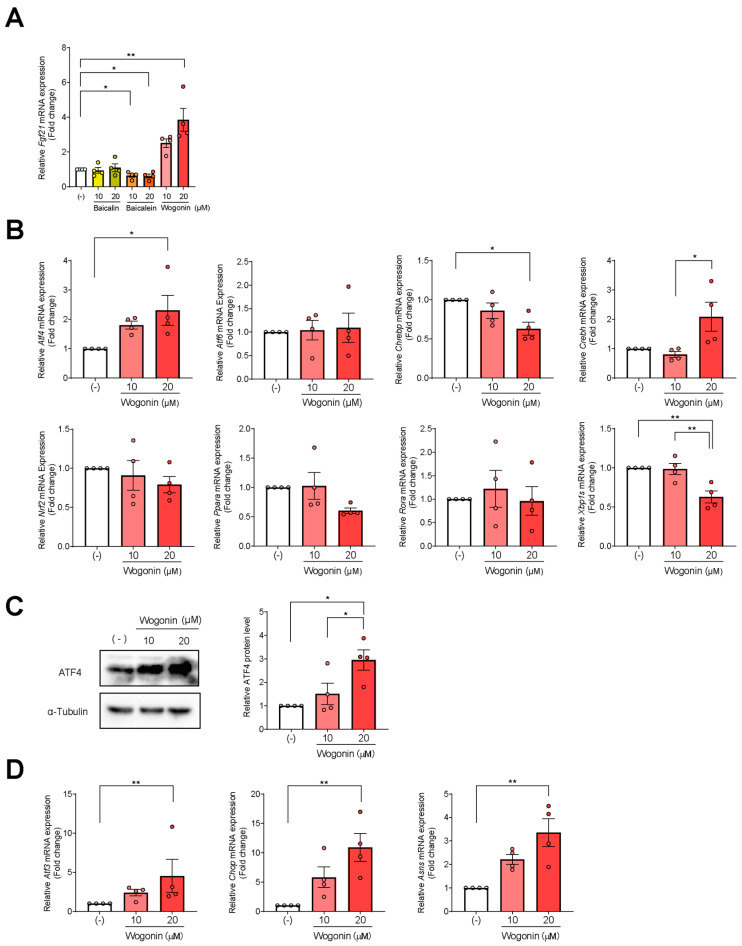
Wogonin induces *Atf4* and *Fgf21* expression. (**A**) Wogonin increased *Fgf21* expression in AML12 cells. Cells were treated with 10 and 20 µM of baicalin, baicalein, and wogonin for 48 h. *n* = 4 per group. (**B**) Gene expression of FGF21-regulating transcription factors in AML12 cells. Cells were treated with 10 and 20 µM of wogonin for 48 h. *n* = 4 per group. (**C**) Wogonin increased the protein levels of ATF4 in AML12 cells. Cells were treated with 10 and 20 µM of wogonin for 48 h. The protein bands were quantified. *n* = 4 per group. (**D**) Wogonin increased the expression of genes regulated by ATF4 in AML12 cells. Cells were treated with 10 and 20 µM of wogonin for 48 h. *n* = 4 per group. Data are represented as mean ± SD. * *p* < 0.05; ** *p* < 0.01. Comparisons among multiple groups were assessed using one-way ANOVA, followed by Tukey’s post hoc test.

SBE mainly comprises the flavonoids baicalin, baicalein, and wogonin [35,39]. To identify the major compound(s) from SBE that contribute(s) to its *Fgf21*-enhancing activity, AML12 cells were treated with the predominant SBE flavonoids—baicalein, baicalin, and wogonin—at 10 and 20 µM for 48 h, followed by measuring *Fgf21* expression by qPCR. Wogonin increased *Fgf21* expression in a dose-dependent manner, while baicalein and baicalin showed no effect (Figure 2A). These findings support that wogonin could be a candidate to increase *Fgf21* expression.

### 3.3. ATF4 Increased Fgf21 Expression in Response to Wogonin

*Fgf21* expression is up-regulated by transcription factors such as ATF4, ATF6, ChREBP, CREBH, NRF2, PPARα, retinoic acid-related orphan receptor α (RORα), and XBP1s. To identify the master transcription factor controlling wogonin-mediated *Fgf21* expression, we examined the gene expression of these factors. Wogonin increased *Atf4* expression alone in a dose-dependent manner; *CrebH* expression was enhanced only at high doses of wogonin (Figure 2B). The expression of the other transcription factors was either unchanged or decreased in response to wogonin (Figure 2B). These results support that wogonin would regulate *Fgf21* expression via ATF4. Consistent with *Atf4* mRNA levels, wogonin increased ATF4 protein levels (Figure 2C) as well as the expression of some typical target genes of ATF4, such as *Atf3*, asparagine synthetase (*Asns*), and CCAAT-enhancer-binding protein homologous protein (*Chop*), in a dose-dependent manner (Figure 2D). These results suggest that ATF4 regulates wogonin-mediated *Fgf21* expression in AML12 cells and confirm that wogonin increases ATF4 transcriptional activity.

### 3.4. Wogonin Controls ATF4 at the Transcription Level

**Figure 3:**
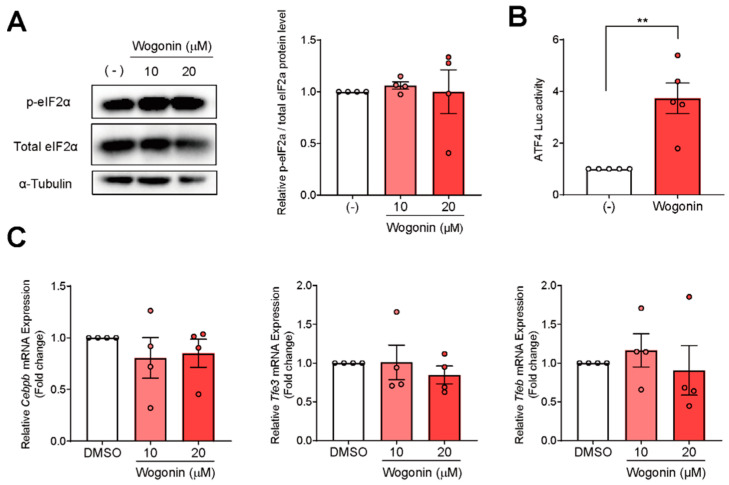
Wogonin affects the promoter activity of *Atf4*. (**A**) The protein levels of phospho- and total-eIF2α were not changed in AML12 cells. Cells were treated with 10 and 20 µM of wogonin for 48 h. The protein bands were quantified. *n* = 4 per group. (**B**) Wogonin increased ATF4-luciferase activity. AML12 cells were co-transfected with the pGL3-ATF4 and pRL-SV40 vectors. After 24 h of transfection, cells were treated with 20 μM of wogonin for 24 h. *n* = 5 per group. Data are represented as mean ± SD. (**C**) The gene expression of ATF4-regulating transcription factors in AML12 cells. Cells were treated with 10 and 20 µM of wogonin for 48 h. *n* = 4 per group. Data are represented as mean ± SD. ** *p* < 0.01. Comparisons between two groups were assessed using unpaired two-tailed *t* tests and those among multiple groups were assessed using one-way ANOVA, followed by Tukey’s post hoc test.

*Atf4* expression is controlled by the PERK-eIF2α signaling pathway. ER stress induces PERK, which activates eIF2α by phosphorylating it, eventually inducing the translation of ATF4. Therefore, we determined the protein levels of phospho-eIF2α (p-eIF2α) and total eIF2α by Western blotting. We found that wogonin did not elevate the levels of either p-eIF2α or total eIF2α (Figure 3A), suggesting that its stimulatory effect on ATF4 expression was not mediated via eIF2α signaling. To determine whether wogonin activates *Atf4* promoter activity, we performed a luciferase assay with pGL3-ATF4 vector containing –0.5 kbp to –100 bp of the mouse *Atf4* promoter. Wogonin significantly increased the luciferase activity inside pGL3-ATF4-transfected AML12 cells (Figure 3B). Taken together, these results indicate that wogonin enhanced *Atf4* expression by acting on its promoter. Concerning the transcriptional regulation of ATF4, *Atf4* expression has been shown to be up-regulated by NRF2 [21], TFE3, and TFEB [22], and down-regulated by C/EBPβ [23]. However, the expressions of these genes were unchanged after wogonin treatment (Figure 3C), indicating that the known *Atf4*-regulating transcription factors are not involved in wogonin’s mode of action.

### 3.5. Knockdown of Atf4 Suppresses Wogonin-Induced Fgf21 Expression

**Figure 4:**
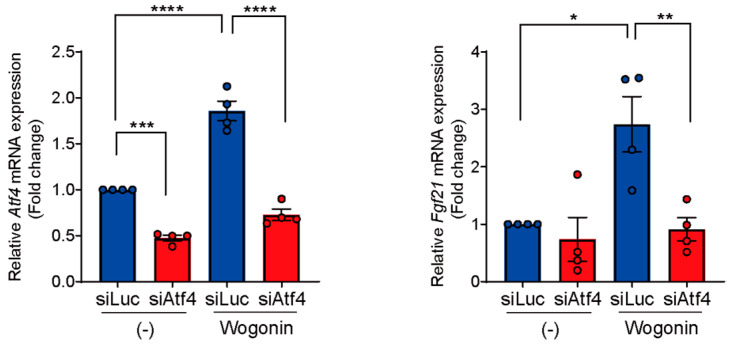
Deficiency of ATF4 suppresses wogonin-induced *Fgf21* expression. *Fgf21* expression was suppressed by transfecting AML12 cells with siRNA against ATF4. After 24 h of transfection, cells were treated with 20 µM of wogonin for 48 h. *n* = 4 per group. Data are represented as mean ± SD. * *p* < 0.05; ** *p* < 0.01; *** *p* < 0.001; **** *p* < 0.0001. Comparisons among multiple groups were assessed using one-way ANOVA, followed by Tukey’s post hoc test.

To confirm the necessity of ATF4 for wogonin-induced *Fgf21* expression, we performed a loss-of-function analysis by knocking down ATF4 using siRNA (siAtf4) in AML12 cells. AML12 cells were transfected with siRNAs and then treated with wogonin. We confirmed that siAtf4 efficiently reduced *Atf4* expression with/without wogonin (Figure 4). ATF4 knockdown eliminated wogonin-induced *Fgf21* expression (Figure 4), demonstrating that ATF4 plays a crucial role in wogonin’s effects on *Fgf21*.

## 4. Discussion

FGF21 is mainly secreted by the liver, and it systemically improves nutrient metabolism with paracrine action; thus, finding a drug that induces FGF21 in the liver is important. Our study identified that wogonin, a component of SBE, activates *Fgf21* expression in AML12 cells—a process mediated by the transcription factor ATF4—by using the WAKANYAKU library.

*S. baicalensis* has been traditionally used as a medicinal herb in Asia, including Japan, China, and Korea. SBE is broadly used for the clinical treatment of hyperlipidemia, atherosclerosis, hypertension, and inflammatory diseases [29]. SBE suppresses sterol regulatory element binding protein-1c (SREBP-1c) activity by down-regulating *Srebf1c* expression and activates AMPK in the liver, improving non-alcoholic fatty liver disease [32]. In type 2 diabetic db/db mice, SBE also activates AMPK activity and improves metabolic disorders in the liver, ameliorating this disease [33]. SBE enhances the activity of metformin, a drug for treatment of type 2 diabetes, activates AMPK in type 1 diabetes mice and streptozotocin (STZ)-induced diabetic rats [34]. SBE contains flavonoids, such as baicalin, baicalein, and wogonin, which exert anti-obesity and antihyperlipidemic effects [40]. It has been reported that baicalin and baicalein activates AMPK [32,41]. The effect of wogonin on AMPK activation remains unknown. Wogonin activates PPARα, ameliorating the diabetic phenotypes in *db*/*db* mice [36]. Study of the molecular mechanism underlying the improvement effects of SBE on metabolic disorders is still insufficient. FGF21, a master regulator for metabolic homeostasis, is focused on the treatment of metabolic disorders. Baicalein has been reported to increase *Fgf21* expression in C2C12 myotubes by activating the transcription factor RORα [42]. However, whether SBE itself can up-regulate *Fgf21* expression has not been reported yet. Here, we found that SBE and one of its main components, wogonin, increased *Fgf21* expression in AML12 cells, while baicalein and baicalin did not. As the liver is the main organ to secrete FGF21, wogonin might be a therapeutic that can ameliorate metabolic disorders. This could explain the multiple effects of SBE on metabolic disorders.

*Fgf21* expression, critically regulated by PPARα and CREBH, is induced in the liver during fasting [43]. Wogonin activates PPARα in the liver and adipose tissues [36]. However, the gene expression of these transcription factors was unchanged in wogonin-treated cells. Additionally, the expression of other known *Fgf21* regulators, such as ATF6, ChREBP, NRF2, RORα, and XBP1s, was unchanged. The only factor whose gene expression was enhanced by wogonin was ATF4. Knockdown of ATF4 blunted wogonin-induced *Fgf21* expression, confirming its essential role in wogonin’s mechanism of action.

ER stress has three branches that increase *Fgf21* expression: (1) ATF6, (2) Inositol-requiring enzyme 1–XBP1, and (3) PERK–ATF4 [13]. During ER stress, the translation of ATF4 is activated by PERK-mediated eIF2α phosphorylation [44]. eIF2α is also phosphorylated by the kinases other than PERK including PKR, hemeregulated inhibitor (HRI), and general control nonderepressible 2 (GCN2). These kinases are activated in response to different stresses: PKR by the infection with certain viruses, HRI by the limitation of heme, GCN2 by the deprivation of essential amino acids [45]. These stimuli finally converge on eIF2α phosphorylation and induce *Atf4* expression. However, wogonin-induced ATF4 activation was not mediated by this pathway, as seen by the unchanged levels of p-eIF2α after wogonin treatment. Wogonin activates ATF4 without activating ER stress pathways. Additionally, wogonin did not up-regulate either *Atf6* or *Xbp1s*. Taken together, wogonin cannot activate ER stress pathways. C/EBPβ has been identified as a suppressor of *Atf4* expression [23], while TFE3, TFEB, and NRF2 have been identified as activators [21]. However, the expression of these molecules was unchanged as well upon wogonin treatment. Thus, the mechanism of wogonin-induced *Atf4* expression remains a mystery that needs to be investigated in future studies.

Chronic inflammation underlies metabolic syndromes, including obesity, diabetes, hyperlipidemia, and high blood pressure [46]. Wogonin has displayed anti-inflammatory activity in several animal models, including lipopolysaccharide-induced acute liver injury, acute lung injury, and kidney injury [47]. Wogonin activates the expression of PPARγ and subsequently suppresses the nuclear factor-κB pathway [47]. Besides these effects, wogonin has been shown to ameliorate metabolic diseases [48]. In this study, we revealed that wogonin can induce *Fgf21* expression in AML12 cells, which are derived from the liver, the main FGF21-secreting organ. FGF21 can improve the status of various metabolic diseases; thus, our findings present wogonin as an attractive therapeutic for metabolic syndromes.

## Figures and Tables

**Table 1:** Primers used for real-time PCR analysis.

Gene	Forward Primer (5′ to 3′)	Reverse Primer (5′ to 3′)
*Asns*	TTACCTGTCTCTGCCGCCAGAT	CACTGAAGGCTTCTTTGGGTCG
*Atf3*	TTTGCTAACCTGACACCCTTTG	AGAGGACATCCGATGGCAGA
*Atf4*	CCTGAACAGCGAAGTGTTGG	TGGAGAACCCATGAGGTTTCAA
*Atf6*	GGACGAGGTGGTGTCAGAG	GACAGCTCTTCGCTTTGGAC
*Cebpb*	TACGAGCCCGACTGCCTG	TCGGAGAGGAAGTCGTGGTG
*Chop*	GGAGGTCCTGTCCTCAGATGAA	GCTCCTCTGTCAGCCAAGCTAG
*Chrebp*	AATGGGATGGTGTCTACCGC	GGCGAAGGGAATTCAGGACA
*Fgf21*	GGCAAGATATACGGGCTGAT	TCCATTTCCTCCCTGAAGGT
*CrebH*	AGATCAGGGAGGATGGAACA	TCAAAGTGAGGCGATCCATA
*Cyclophilin*	TGGCTCACAGTTCTTCATAACCA	ATGACATCCTTCAGTGGCTTGTC
*Nrf2*	CAAGACTTGGGCCACTTAAAAGAC	AGTAAGGCTTTCCATCCTCATCAC
*Ppara*	ACGCGAGTTCCTTAAGAACCTG	GTGTCATCTGGATGGTTGCTCT
*Rora*	GATGACCTCAGCACCTATATGGA	CGGGTTTGATCCCATTGATGTC
*Tfe3*	AGGATCAAAGAGCTGGGCAC	CCGGCTCTCCAGGTCTTTG
*Tfeb*	CAGAAGCGAGAGCTAACAGAT	TGTGATTGTCTTTCTTCTGCCG
*Xbp1s*	CTGAGTCCGAATCAGGTGCAG	GTCCATGGGAAGATGTTCTGG

## Data Availability

Not applicable.

## Supplementary Materials

The following supporting information can be downloaded at: https://www.mdpi.com/article/10.3390/nu14193920/s1, Table S1: A library consisting of 122 herbal extracts.
